# Energy Management Strategy Based on a Novel Speed Prediction Method

**DOI:** 10.3390/s21248273

**Published:** 2021-12-10

**Authors:** Jiaming Xing, Liang Chu, Zhuoran Hou, Wen Sun, Yuanjian Zhang

**Affiliations:** 1 State Key Laboratory of Automotive Dynamic Simulation and Control, Jilin University, Changchun 130021, China; xingjm19@mails.jlu.edu.cn (J.X.); chuliang@jlu.edu.cn (L.C.); houzr20@mails.jlu.edu.cn (Z.H.); 2 College of Automotive Engineering, Changzhou Institute of Technology, Changzhou 213032, China; sunw@czu.cn; 3 School of Mechanical and Aerospace Engineering, Queen’s University Belfast, Belfast BT9 5AG, UK

**Keywords:** speed prediction, deep learning, energy management strategy, model predictive control

## Abstract

Vehicle speed prediction can obtain the future driving status of a vehicle in advance, which helps to make better decisions for energy management strategies. We propose a novel deep learning neural network architecture for vehicle speed prediction, called VSNet, by combining convolutional neural network (CNN) and long-short term memory network (LSTM). VSNet adopts a fake image composed of 15 vehicle signals in the past 15 s as model input to predict the vehicle speed in the next 5 s. Different from the traditional series or parallel structure, VSNet is structured with CNN and LSTM in series and then in parallel with two other CNNs of different convolutional kernel sizes. The unique architecture allows for better fitting of highly nonlinear relationships. The prediction performance of VSNet is first examined. The prediction results show a RMSE range of 0.519–2.681 and a R2 range of 0.997–0.929 for the future 5 s. Finally, an energy management strategy combined with VSNet and model predictive control (MPC) is simulated. The equivalent fuel consumption of the simulation increases by only 4.74% compared with DP-based energy management strategy and decreased by 2.82% compared with the speed prediction method with low accuracy.

## 1. Introduction

With the development of technology, speed prediction has been widely used in economic, industry and transportation [1,2,3,4,5,6]. In the vehicle industry, speed prediction has become an important part of intelligent vehicle energy management strategies [7]. As one of the innovative control architectures, hierarchical control architectures equipped with prediction modules have an indispensable contribution in modern control theory [8,9]. In the hierarchical control architecture of new energy vehicles (NEVs), the upper control architecture is mainly responsible for the acquisition of prior information. The vehicle speed prediction, as an effective method to obtain information about future driving conditions, has a direct impact on the control effect of the bottom control [4]. Therefore, the accuracy and efficiency of prediction affect the performance and practicality of the energy management system. Improving the accuracy of vehicle speed prediction will be the main research content of this paper.

The accuracy of speed prediction affects the optimization effect of energy management strategies. In the terms of instantaneous-optimization-based energy management strategies, Jiazheng PEI used six different speed prediction methods to construct model prediction control (MPC) energy management strategies, respectively. The results showed that the method with smaller speed prediction error obtained better fuel economy [10]. In the terms of global-optimization-based energy management strategies, dynamic programming (DP), which belongs to the global-optimization-based energy management strategies, requires prior information of the vehicle driving conditions before optimizing the optimal control scheme [11]. Prior information and heavy computation pose difficulties for its application in real-time control. To remedy this deficiency, Menglin Li combined speed prediction and DP. The driving cycle was divided into finite intervals and the speed prediction method of deep neural network was used to solve in segments within intervals. The results showed that the speed prediction accuracy could be improved to reduce the fuel consumption by 3.34% per 100 km [12]. The higher the accuracy of speed prediction, the better the optimization effect.

One of the challenge in studying energy management strategies for hybrid electric vehicle (HEV) is improving the accuracy of vehicle speed prediction [13]. With the development of speed prediction algorithms, more and more methods are available. The commonly used methods for speed prediction are linear regression analysis [14,15], vehicle dynamics model prediction [16,17], stochastic prediction [18,19] and machine learning [20,21,22,23].

Linear regression analysis is a statistical analysis method to determine the quantitative relationship between two or more variables [24]. Based on the availability of extensive observational data, mathematical and statistical methods are used to establish the regression relationship between vehicle speed and its influence factors. Mirbaha, B et al. selected traffic and weather data of a two-lane highway located in northwestern Iran for their analysis. A linear regression model was used for speed prediction and analysis of the correlation between the average vehicle speed and variables including vehicle flow, percentage of heavy vehicles, rainfall, etc. [14]. Maji, A et al. used a multiple linear stepwise regression method to develop 85th percentile and 98th percentile speed prediction models for four-lane horizontal curves on rural roads and obtained a minimum Root Mean Square Error (RMSE) of 4.37 km/h [15]. The regression analysis method has a simple model with poor robustness, which cannot fit highly nonlinear relationships well.

The vehicle dynamics model prediction method is based on a vehicle dynamics model that calculates the vehicle acceleration by analyzing the wheel forces to evaluate the vehicle speed. Li et al. built a vehicle controller by predicting the maximum vehicle speed based on the vehicle lateral dynamics when entering a curve. The controller provided an energy management strategy that improved the safety and fuel economy compared with equivalent consumption minimization strategy [16]. Ma, Z et al. proposed a novel method to identify the normal load of each tire in a heavy truck in order to estimate the longitudinal velocity. The simulation showed that the predicted velocity could follow the trend of the real velocity and no significant lag was found [17]. Since the vehicle dynamics model needs to consider various parameters such as turning radius, vehicle mass, tire stiffness, etc., the model is simplified compared with the actual vehicle in order to reduce the modeling difficulty. The speed prediction accuracy is limited by the model accuracy and simplification conditions.

As an important method for stochastic prediction, Markov chains are widely used [25,26]. Markov chain is a stochastic data-driven model that utilizes the state transfer matrix and the current state to predict the future driving state. Jaewook shin et al. proposed a vehicle speed prediction algorithm based on a Markov chain model with speed constraints. The algorithm was experimentally verified to provide a mean square error of 3.8041 km/h [18]. Karbowski et al. used Markov chains to predict the future speed of the vehicle and designed an energy management strategy for plug-in hybrid vehicles. Fuel savings of up to 5.7% were achieved on a 36 km route in Munich [19]. The Markov chain method captures vehicle speed to construct a state transfer matrix for speed prediction. The speed variation is more in line with the real driving conditions of the vehicle. However, little historical information is considered and stochastic prediction leads to large fluctuations in vehicle speed.

The methods described above have simpler prediction models with fewer adjustable parameters, which means weaker fitting of nonlinear relationships and poorer accuracy in predicting vehicle speeds. The development of machine learning techniques has brought new solutions for vehicle speed prediction. Machine learning can learn features from a limited amount of observed data, utilize the features to predict output and has a better nonlinear fitting capability [20,27,28]. Machine learning is divided into shallow learning and deep learning. Traditional shallow learning is weak in feature extraction and requires effort for data processing. If the features are not properly selected, there is no guarantee that there is a stable mapping relationship between the input and output of the sample so that the prediction effect is seriously affected [29,30]. Deep learning differs from traditional shallow learning in that: (1) it emphasizes the depth of the model architecture and improves the nonlinear fitting ability by setting a large number of parameters; (2) it explicitly highlights the importance of feature processing by transforming the feature in the original space to a new feature space through layer-by-layer feature transformation, which makes classification or prediction easier and does not depend on expert experience. In recent years, convolutional neural network (CNN), a powerful deep learning method, has been widely used in prediction due to its excellent feature extraction capability [21,31,32]. Loaiza, F.A. et al. presented a CNN method for speed prediction of a large-scale traffic network. The empirical results showed that the method reduced the convergence time without losing the performance of the predictions [33]. Ma, X. et al. proposed a CNN method that learns traffic as images and predicts large-scale, network-wide traffic speed. The results showed that the CNN outperformed other algorithms on testing data with an average accuracy improvement of 42.91% [34]. CNN reduces the number of parameters to be adjusted by neural networks through perceptual field and weight sharing, minimizes the preprocessing requirements of data and achieves high accuracy vehicle speed prediction. However, CNN is not designed to handle temporal data specifically. For vehicle speeds with time-series characteristics, the prediction accuracy of CNN is subject to loss.

Compared with CNN, long short-term memory (LSTM) neural network with long sequence processing capability has good performance in sequence prediction to detect long-term dependencies [35]. Hochreiter and Schmidhuber proposed LSTM in 1997 [36]. It has been refined by many researchers in the following decade [37,38,39]. In the field of transportation, LSTM is one of the most commonly used prediction models. Ma et al. pioneered the introduction of LSTM for vehicle speed prediction and found that the LSTM neural network achieved the best results in terms of prediction accuracy and stability by comparing it with other neural networks [22]. Yeon, K. et al. used information from the past 30 s to predict the future vehicle speed based on LSTM. The results showed that LSTM has better prediction performance compared with CNN [23]. LSTM gets an edge on sequence problem and has long time memory capability. However, except for the temporal features, it has a poor ability to extract the remaining features.

The complexity of machine learning allows for a stronger nonlinear fitting capability. A high accuracy model for speed prediction can be obtained by training a large amount of data. However, a general machine learning model that solves a series of complex problems does not exist. Individual model always has certain limitations in application.

In the context, this paper combines CNN and LSTM to propose a novel neural network structure based deep learning specifically for vehicle speed prediction, named VSNet. VSNet can identify the mapping relationship between vehicle signals and vehicle speed to accurately predict the future vehicle speed. In this paper, VSNet is compared with Markov chain combined with Monte Carlo (MCMC), support vector machine (SVM) and CNN to verify the effectiveness of VSNet in predicting vehicle speed. Finally, for a parallel HEV, the four speed prediction methods are combined with MPC for simulation experiments to verify the impact of prediction accuracy on MPC.

The innovations of this paper are as follows.

(1) The temporal vehicle signals are arrayed as fake images, which are adopted as the input sample of VSNet. Three types of convolutional kernels with different sizes are used to extract the temporal features, vehicle signal features and comprehensive features in a single sample, respectively.
(2) Generate sample sequences based on time series. LSTM is used to extract the sequential features between the sample.
(3) The combination of CNN and LSTM is used to construct VSNet, which makes up for the defects of the individual model and enhances the learning ability.

The remainder of this paper is structured as follows. Section 2 introduces the deep learning method and proposes VSNet. Section 3 introduces the vehicle model and MPC model. Section 4 shows the speed prediction results and combines MPC with speed prediction methods for vehicle simulation validation. Section 5 shows the conclusion and future outlook.

## 2. Speed Prediction Method

**Figure 1:**
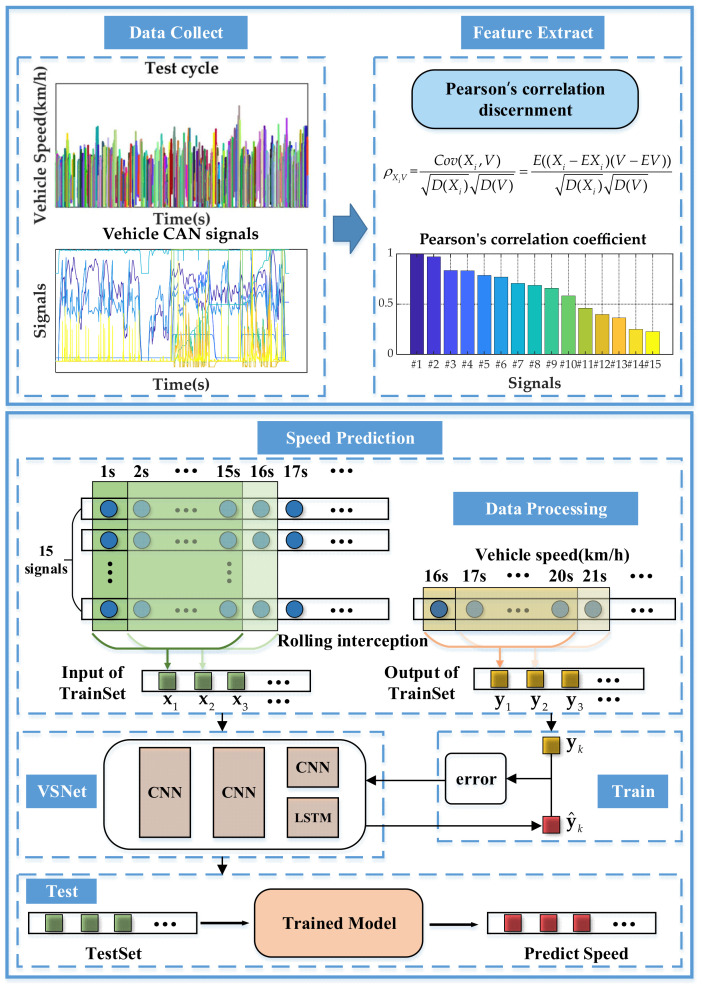
The flow chart of speed prediction.

Road experiments are conducted to collect raw data of a vehicle. By calculating the Pearson correlation coefficient between each signal and vehicle speed, the top 15 signals that have the strongest correlation with vehicle speed are selected for feature extraction. The fake image is obtained by fixed-step interception and the training set is obtained by rolling interception. The prediction model of vehicle speed is obtained by training the parameters offline through error back propagation. When the vehicle is in motion, the vehicle speed can be predicted online in real time by simply inputting the vehicle information into the trained model. The flow chart of speed prediction is shown by Figure 1.

### 2.1. Data Processing

In this paper, road experiments are performed on a hybrid electric vehicle to collect data of signals. The data from each signals forms a vector that changes over time. Vectors need to be arranged into a matrix as a fake image to be input to VSNet. To ensure the reasonableness of fake images, selected signals need to be able to establish a mapping relationship with vehicle speed. In order to make a mapping between input data and predicted vehicle speed, this paper uses Pearson correlation to judge the relationship between each vehicle signal and vehicle speed and selects fifteen signals for subsequent research, which ensures data quality and reduces the input data scale.

**Figure 2:**
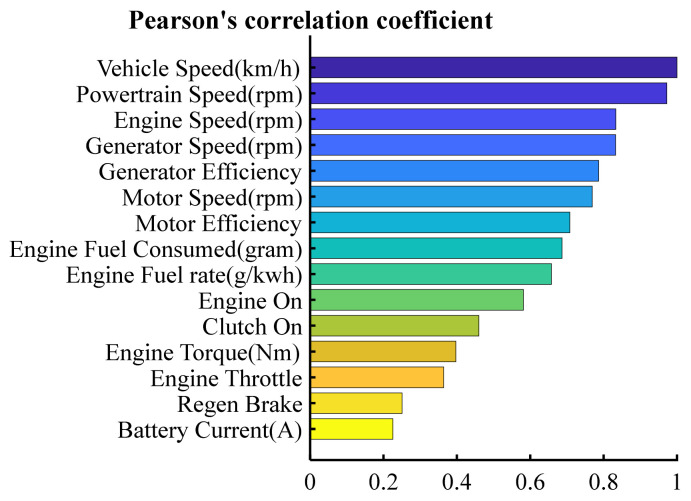
The illustration of Pearson correlation coefficient.

Pearson correlation is a method proposed by the British statistician Pearson in the 20th century to calculate the linear correlation [40]. The Pearson correlation coefficient between vehicle signal and vehicle speed can be calculated by

(1) $$\rho_{X_{i}V}=\frac{Cov(X_{i},V)}{\sqrt{D(X_{i})}\sqrt{D(V)}}=\frac{E((X_{i}-EX_{i})(V-EV))}{\sqrt{D(X_{i})}\sqrt{D(V)}}$$

where E denotes expectation, D denotes variance, Cov denotes covariance, V denotes vehicle speed and $X_{i}$ denotes the ith vehicle signal. The selected signals and their Pearson correlation coefficients with vehicle speed are shown by Table 1 and Figure 2.

Samples used for training model consists of input and output. The input of samples is obtained by intercepting the selected vehicle signal sequences with sampling time of 15 s and rolling time of 1 s. The output of samples is obtained by intercepting the vehicle speed sequence with sampling time of 5 s and rolling time of 1 s. The format of samples is described by Equations (2) and (3).

(2) $$x_{k}=\left\{\begin{array}{cccc} S_{1}^{k-14} & S_{1}^{k-13} & \cdots & S_{1}^{k}\\ S_{2}^{k-14} & S_{2}^{k-13} & \cdots & S_{2}^{k}\\ \vdots & \vdots & \ddots & \vdots \\ S_{15}^{k-14} & S_{15}^{k-13} & \cdots \; & S_{15}^{k} \end{array}\right\}$$

(3) $$y_{k}=\left\{\begin{array}{l} S_{1}^{k+1}\\ S_{1}^{k+2}\\ S_{1}^{k+3}\\ S_{1}^{k+4}\\ S_{1}^{k+5} \end{array}\right\}$$

where k denotes both the time and the sample label; $x_{k}$ and $y_{k}$ are input and output of the kth sample, respectively; $S_{i}$ denotes the ith vehicle signal in Table 1.

### 2.2. CNN

**Figure 3:**
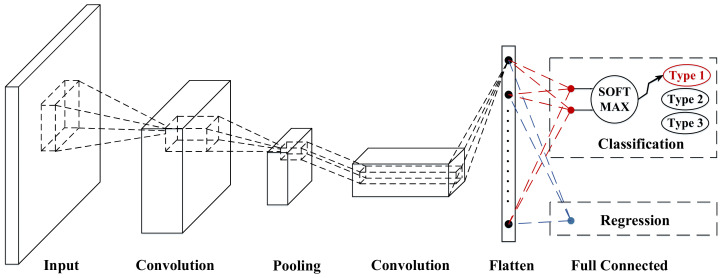
The illustration of a basic CNN.

The data collected in this paper are two-dimensional fake images with vehicle signal dimension and time dimension. Due to the unique structure, CNN has high accuracy regression results and classification results for image processing. Therefore, it is chosen to build speed prediction model. A basic CNN architecture is shown by Figure 3. CNN is a deep feedforward neural network that extracts features of the data within the coverage by a unique convolutional computation [29]. Its structure consists of input layer, intermediate layer and output layer, where the intermediate layer includes convolutional layer, pooling layer, activation function and batch normalization layer.

#### 2.2.1. Convolutional Layer

The role of the convolution layer is compression and purification in order to enhance input signals and reduce noise. The neurons between the convolutional layers are locally connected and share the weights. This design greatly speeds up the training and decreases the number of weights and biases. Local connectivity means that the neurons in the upper layer are connected to the neurons in the lower layer only in a local area, which is also called local receptive field. The mapping from the previous layer to the next layer is called the feature mapping. The weight that defines the feature mapping is called the shared weight. The bias of the feature mapping is called shared bias. Collectively, they are referred to as a convolution kernel. A single convolution kernel can detect only one type of local feature in most cases, so multiple convolution kernels are often needed in order to implement the mapping of multiple features. The rules of convolution operation for multiple input features are shown by Equation (4). Additionally, for the convolution operation with step size d, the formula is described by Equation (5).

(4) $$Z_{i,j,k}^{l+1}=\sum_{s,m,n}A_{s,j+m-1,k+n-1}^{l}W_{i,s,m,n}^{l+1}+B_{i}^{l+1}$$

(5) $$Z_{i,j,k}^{l+1}=\sum_{s,m,n}A_{s,(j-1)d+m,(k-1)d+n}^{l}W_{i,s,m,n}^{l+1}+B_{i}^{l+1}$$

where Z denotes the input of the neuron, l denotes the lth layer, i denotes ith feature vector, j and k denotes the jth row and kth column of a feature vector, respectively, A denotes the output of the neuron, s denotes the number of feature vectors in the layer, m and n denotes the value of (m,n) position in a convolution kernel, and B is the bias.

#### 2.2.2. Pooling Layer

The reason why convolution is used to extract features is that features in one region of the input data are most likely to be present in another region as well. Therefore, it is necessary to further aggregate statistics of features at different regions, which is called pooling. The pooling layer is less prone to overfitting and can reduce the feature resolution. The pooling method used in this paper is maximum pooling, whose equation is described by

(6) $$ap_{i,j,k}^{l}=\underset{m,n}{\max}\{a_{i,(j-1)d+m,(k-1)d+n}^{l}\}$$

where ap denotes the output of the pooling layer, d denotes the step size of the pooling, m and n denotes the value of (m,n) position in a pooling kernel, and the rest of the parameters are the same as before.

#### 2.2.3. Activation Function

The activation function is responsible for mapping the input of a neuron to the output. It is crucial for artificial neural network models to learn and understand complex and non-linear functions. The activation function used in this paper is the ELU function, which performs constant operations on positive inputs and exponential nonlinear operations on negative inputs. Its function expression is described by

(7) $$\mathrm{ELU}(x)=\left\{\begin{array}{cc} x & x\geq 0\\ \alpha (e^{x}-1) & x<0 \end{array}\right.$$

where *x* is the input of the activation layer and α is the adjustable parameter.

#### 2.2.4. Batch Normalization Layer

The most widely used normalization method in convolutional neural networks is batch normalization. Batch normalization has the role in preventing gradient explosion and gradient disappearance. In the backpropagation process of neural network, the gradient of each layer is calculated by multiplying the gradient passed from the upper layer. If the gradient of each layer is close to 0, the more layers are propagated, the smaller the gradient is. This causes the gradient disappearance during the back propagation. On the contrary, if the gradient of each layer is more than 1, it will lead to gradient explosion. The normalized data is mapped by the activation function to keep the gradient at a suitable level, so that the gradient does not disappear or explode during the training back propagation. In the training process, batch size is used to determine the number of samples selected for once training. Assuming that there are m elements in a batch size, the set $B=\{x_{1},\ldots ,x_{m}\}$ is formed. The strategy of batch normalization is to first compute the sample mean and sample variance in turn for B, and then perform normalization, translation and scaling on the sample data. The formulas are described by Equations (8)–(11).

(8) $$\mu_{B}=\frac{1}{m}\sum_{i=1}^{m}x_{i}$$

(9) $$\sigma_{B}^{2}=\frac{1}{m}\sum_{i=1}^{m}(x_{i}-\mu_{B}{)}^{2}$$

(10) $${\hat{x}}_{i}=\frac{x_{i}-\mu_{B}}{\sqrt{\sigma_{B}^{2}+\varepsilon }}$$

(11) $$BN_{\gamma ,\beta }(x_{i})=\gamma {\hat{x}}_{i}+\beta$$

where $\mu_{B}$ is the sample mean, $\sigma_{B}^{2}$ is the sample variance, ε is an infinitesimal positive number, and γ and β are training parameters.

#### 2.2.5. Output Layer

The CNN implements regression through a fully connected layer. The fully connected layer extracts and integrates all useful information through matrix multiplication, which is equivalent to feature space transformation. Together with the nonlinear mapping of the activation function, multiple layers of fully connected layers can theoretically simulate any nonlinear transformation.

### 2.3. LSTM

**Figure 4:**
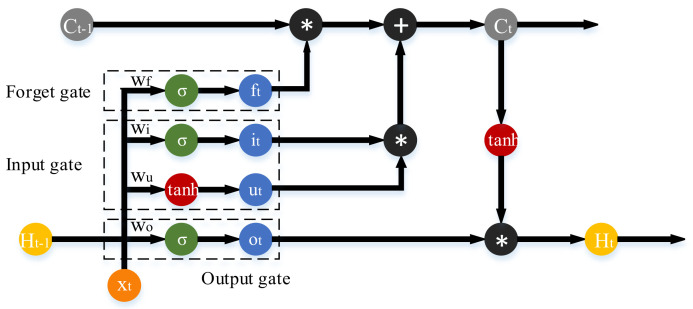
The illustration of a basic CNN.

LSTM is a temporal recurrent neural network suitable for processing and predicting important events in time series with relatively long intervals and delays [39]. The structure of LSTM is shown by Figure 4. LSTM adds a unit called cell that judges whether the information is useful or not. Only information that meets the conditions is remembered, the ones that do not are ignored.

The sigmoid function is applied as a gate to return a value in the interval from 0 to 1. In this way, the proportion of information flowing out is controlled. The expression of the function is described by

(12) $$\sigma (x)=\frac{1}{1+e^{-x}}$$

The tanh function is derived from the basic hyperbolic functions. It returns a value in the interval from −1 to 1 to control the increase or decrease of the information. Its function expression is described by

(13) $$\tanh x=\frac{\sinh x}{\cosh x}=\frac{e^{x}-e^{-x}}{e^{x}+e^{-x}}$$

In LSTM, three sigmoid functions manage three gates, namely forget gate, input gate and output gate. LSTM adds the element of memory state C. The current memory state $C_{t}$ is determined by the portion of the previous memory state $C_{t-1}$ filtered through the forgetting gate plus the portion added in the current period. The portion that is filtered in the previous period depends on the forgetting gate based on the sigmoid function. When the value of the forgetting gate is 0, it means that the previous memory is completely forgotten. While when the value of the forgetting gate is 1, the previous memory is completely retained. The output formula of the forgetting gate is described by

(14) $$f_{t}=\sigma (w_{f}\cdot [H_{t-1},x_{t}])+b_{f}$$

where $H_{t-1}$ is the model output in period t−1, $x_{t}$ is the model input in period t, $w_{f}$ and $b_{f}$ is the weight and bias of the forgetting gate, respectively, and $f_{t}$ is the output of the forgetting gate in period t.

The input gate is used to update the new memories. The sigmoid function controls the proportion of updated information and the tanh function controls the magnitude and direction of the update. The two portions are multiplied to get the new memory of the current period. Then, the portion of the previous memory is added to get the memory state of the current period. The formula is described by Equations (15)–(17).

(15) $$i_{t}=\sigma (w_{i}\cdot [H_{t-1},x_{t}])+b_{i}$$

(16) $$u_{t}=\tanh(w_{u}\cdot [H_{t-1},x_{t}])+b_{u}$$

(17) $$\iota_{t}=i_{t}\cdot u_{t}$$

where $w_{i}$ and $b_{i}$ are the weight and bias of the information update ratio, respectively, $i_{t}$ is the output of the sigmoid function of the input gate in the tth period, $w_{u}$ and $b_{u}$ are the weight and bias of the information update magnitude and direction, $u_{t}$ is the output of the tanh function of the input gate in the tth period and $\iota_{t}$ is the output of the input gate in the tth period.

The sigmoid function of the output gate and the tanh function of the current memory are multiplied to obtain the output $H_{t}$ for the current period. $C_{t}$ and $H_{t}$ will flow cyclically to the next period and participate in the calculation. The formula is described by Equations (18)–(20).

(18) $$o_{t}=\sigma (w_{o}\cdot [H_{t-1},x_{t}])+b_{o}$$

(19) $$c_{t}=c_{t-1}\cdot f_{t}+\iota_{t}$$

(20) $$H_{t}{=o}_{t}\cdot \tanh\left(c_{t}\right)$$

where $w_{o}$ and $b_{o}$ are the weight and bias of the output gate, and $o_{t}$ is the output of the output gate in period t.

### 2.4. The Architecture of VSNet

**Figure 5:**
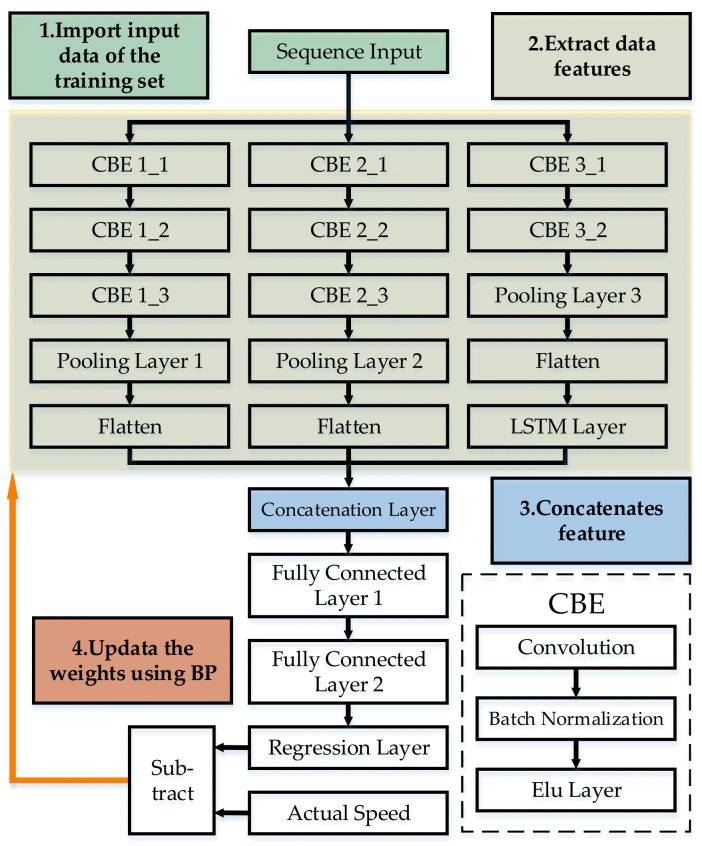
The architecture of VSNet.

The deep learning architecture built in this paper is called VSNet whose architecture is shown by Figure 5. Three different sizes of convolutional kernels are used to extract the features of the samples in parallel. Convolutional kernels of the first size are shaped as a horizontal bar, and extract the sequential features of each vehicle signal in a single sample. As the training progresses, kernels of this size look for a timing variation, which is common to all signals. Convolutional kernels of the second size are shaped as a vertical bar, which tends to extract the relationship features between each vehicle signals related to vehicle speed in a single sample. Convolutional kernels of the third size are a rectangle, which extracts the combined features of signals and sequences. After the process, a LSTM layer is used to extract the sequential features between the sample. Eventually, final results of three types convolution is concatenated by the concatenation layer. The output is performed by a two-layer fully connected neural network to predict the vehicle speed.

The input data format of VSNet is shown by the expression of Equations (2) and (3) above. The input data with time series are combined to constitute the training set, as shown by Equation (21).

(21) $$X=\left\{\begin{array}{l} x_{1}\\ x_{2}\\ x_{3}\\ \vdots \\ x_{n} \end{array}\right\}\;\;\;\;\;\;\;\;\;\;\;\;Y=\left\{\begin{array}{l} y_{1}\\ y_{2}\\ y_{3}\\ \vdots \\ y_{n} \end{array}\right\}$$

where X and Y are the input and output of the training set, respectively.

In the initial stage of model training, the initial weights $W_{0}$ of the model is set randomly. After inputting the training set, the model brings X and W into the calculation for speed prediction, as shown by

(22) $$\hat{Y}=VSNet(X,W)=\left\{\begin{array}{l} {\hat{y}}_{1}\\ {\hat{y}}_{2}\\ {\hat{y}}_{3}\\ \vdots \\ {\hat{y}}_{n} \end{array}\right\}$$

where $\hat{Y}$ is the set of prediction results. The prediction results are compared with the actual output results Y to verify the model prediction accuracy, as shown by

(23) $$S=\sum_{i=1}^{n}{\left(y_{i}-{\hat{y}}_{i}\right)}^{2}$$

where S is the sum of the squared errors. The goal of training is to minimize S by continuously adjusting W. The W is adjusted using the back propagation method and the adjustment magnitude of each weight is calculated by gradient descent method. The calculation formula is shown by

(24) $$\Delta w_{j}=-\eta \frac{\partial S}{\partial w_{j}}$$

where $\Delta w_{j}$ is the step size of once adjustment of the weight and η is the learning coefficient. In order to find the minimal value of S, it is necessary to correct W gradually from its initial value to the value where the partial derivative of S is 0. The chain rule is used to solve the partial derivative of the complex function S. The error is propagated from the output layer by layer and no intermediate steps can be skipped in this process.

$W_{0}$ tends to produce a large error. The calculation based on its derivative also results in a large initial step size. As the error function is adjusted so that S gradually converges to a minimal value, the absolute value of the derivative gradually converges to 0 and the step size is continuously reduced. If η is set too small, the learning speed is too slow and takes too long. On the contrary, if η is set too large, it is very likely to skip the extreme point and continue to oscillate without convergence. Therefore, η is adopted 1.5 × 10^−3^ in this paper to improve the learning speed while ensuring convergence to the extremum.

The update of weights in VSNet is completed in this way.

## 3. Model

In this paper, a HEV is modeled in Simulink platform and a MPC-based energy management strategy is built.

### 3.1. Vehicle Model

**Figure 6:**
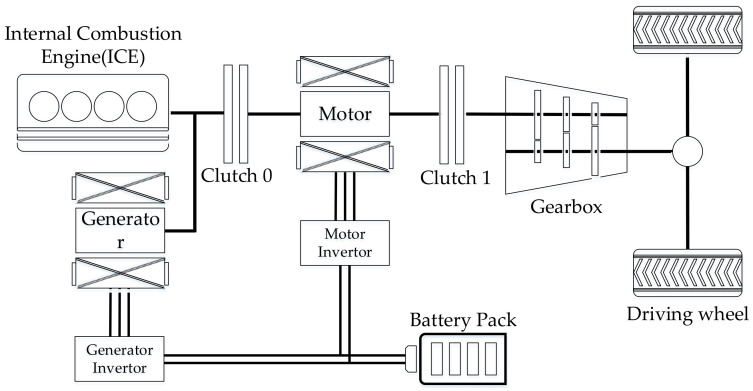
The schematic of the PHEV configuration.

The vehicle model is a parallel HEV. According to the placement of the electrification component, the vehicle belongs to the P2 type, i.e., the motor is placed behind the clutch 0 and before the transmission. The vehicle configuration is shown by Figure 6. When the clutch 0 is disengaged, the motor drives the vehicle alone, avoiding engine operating in the inefficiency area and allowing for brake energy recovery. When the clutch 0 is engaged, the engine and motor work together. According to the power distribution relationship between the engine and the motor, the vehicle driving model can be divided into motor traction model, engine traction mode, charging mode and hybrid traction mode. The switching conditions between modes are mainly determined by the overall vehicle demand power and the state of charge (SOC). Vehicle and component parameters are shown by Table 2.

### 3.2. Model Predictive Control

MPC, as a control method that allows rolling optimization, is widely used in industrial control processes [41]. The mechanism of MPC is described below. At each sampling moment, a finite stages open-loop optimization problem is solved online based on the obtained current measurement information. The first element of the obtained control sequence is applied to the controlled object. At the next stage, the optimization problem is refreshed and solved again by using the new measurements as the initial conditions for predicting the future dynamics of the system.

#### 3.2.1. State Update

**Figure 7:**
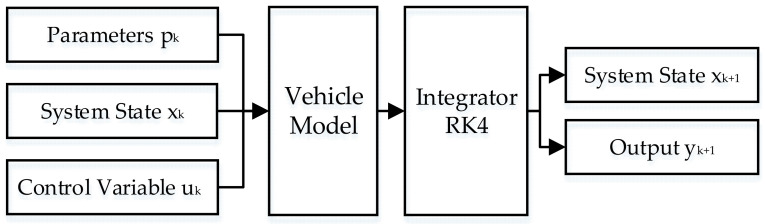
Illustration of the status update process.

Predicting the future state of the system, as shown by Figure 7. The system variables, given control variable and parameters are input at each stage. The system variables and outputs for the next stage can be calculated by combining the input with the vehicle model. The system variables, control variable and parameters are defined as shown by Equation (25). The differential equation of the system is described in Equation (26).

(25) $$\left\{\begin{array}{c} x={\left[v,a,Q_{m},Q_{e}\right]}^{T}\\ u=\lambda \\ p={\left[v^{p},gear\right]}^{T} \end{array}\right.$$

(26) $$\left\{\begin{array}{l} x\prime =f(x,u,p,t)\\ y=g(x,u,p,t) \end{array}\right.$$

where x is the system variable, v is vehicle speed, a is acceleration, $Q_{m}$ is motor energy consumption, $Q_{e}$ is engine energy consumption, u is the control variable, λ is power distribution ratio, p is parameter, $v^{p}$ is target speed and Gear is the gear of the transmission. The vehicle equation constraint for the objective function is shown by

(27) $$\left\{\begin{array}{c} \frac{dv}{dt}=a\\ \frac{da}{dt}=\frac{a_{k}-a_{k-1}}{step}\\ \frac{dQ_{m}}{dt}=P_{m}\\ \frac{dQ_{e}}{dt}=P_{e} \end{array}\right.$$

where $a_{k}$ is the acceleration of the kth stage, step is the step size of the integration, $P_{m}$ is the motor power and $P_{e}$ is the engine power.

In order to solve the differential equations, the fourth-order Longacurta method is used in this paper, which has high accuracy, converges faster and does not require the calculation of higher order derivatives [42]. The calculation formula is shown by

(28) $$\left\{\begin{array}{l} x_{k+1}=x_{k}+\frac{K_{1}}{6}+\frac{K_{2}}{3}+\frac{K_{3}}{3}+\frac{K_{4}}{6}\\ K_{1}=hf(x_{k},t_{k})\\ K_{2}=hf(x_{k}+\frac{K_{1}}{2},t_{k}+\frac{h}{2})\\ K_{3}=hf(x_{k}+\frac{K_{2}}{2},t_{k}+\frac{h}{2})\\ K_{4}=hf(x_{k}+K_{3},t_{k}+h) \end{array}\right.$$

where k is the current stage, f denotes the vehicle model, t is the time, and h is the integration step.

#### 3.2.2. Optimization

**Figure 8:**
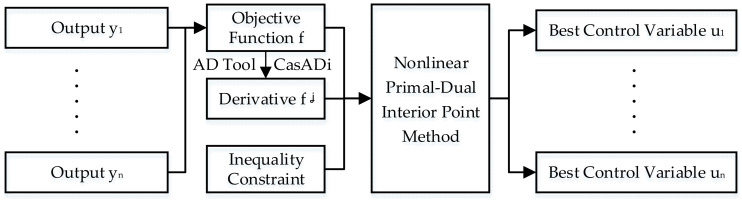
Illustration of the optimization process.

Solving the open-loop optimization problem, as shown by Figure 8. The control variable and parameters in the given time domain can continuously update the state variables of the system to obtain the output sequence in the time domain. The output sequence is accumulated to calculate the optimization objective function. The automatic derivative (AD) tool CasADi is used to find the partial derivatives of the objective function with respect to the control variable [43]. The control variable is solved optimally by combining the inequality constraints of the control variable and vehicle components. The optimization method used is a nonlinear primal-dual interior point method, which solves for the optimal sequence of control variable at each moment of the predicted time domain [44].

(29) $$\min f=\min\sum_{i=1}^{n}y_{i}=\min\sum_{i=1}^{n}[\alpha {\left(v_{i}-v_{i}^{p}\right)}^{2}+\beta Q_{e}+\chi Q_{m}]$$

where f is the objective function, n is horizon; $y_{i}$ is the output of the ith stage, $v_{i}$ is the vehicle speed of the ith stage, $v_{i}^{p}$ is the target vehicle speed of the ith stage, α, β and χ are the scale factor of each item, and $Q_{e}$ and $Q_{m}$ are energy consumption of engine and motor, respectively.

#### 3.2.3. Constraints

The inequality constraints are set up in order to keep the vehicle and its components in a reasonable state and ensure that the results are in the feasible domain. The inequality constraints for the objective function are shown by

(30) $$\left\{\begin{array}{c} -1\leq \lambda \leq 1\\ -1\leq pedal\leq 1\\ -T_{m,min}^{n}\leq T_{m}^{n}\leq T_{m,max}^{n}\\ \begin{array}{c} -T_{e,min}^{n}\leq T_{e}^{n}\leq T_{e,max}^{n}\\ -n_{m,min}\leq n_{m}\leq n_{m,max}\\ -n_{e,min}\leq n_{e}\leq n_{e,max} \end{array} \end{array}\right.$$

where pedal indicates the load of pedal, $T_{m}^{n}$ and $T_{e}^{n}$ are the torque of motor and engine at the rotate speed of n, respectively, $n_{m}$ and $n_{e}$ are the rotate speed of motor and engine, respectively, $T_{m,max}^{n}$, $T_{e,max}^{n}$, $n_{m,max}$ and $n_{e,max}$ are the maximum value of the parameters, respectively, $T_{m,min}^{n}$, $T_{e,min}^{n}$, $n_{m,min}$ and $n_{e,min}$ are the minimum value of the parameters, respectively.

After determining the constraints and objective function, the solution can be solved by the optimizer. The first element of the optimization solution is applied to the system to complete the calculation of MPC at the current moment and obtain the real vehicle state at the next moment. Finally, the optimization problem is refreshed and the control strategy for the next moment is solved again based on the new information.

## 4. Results and Validation

Speed prediction can obtain the future vehicle driving state in advance, which is helpful for energy management strategy to make more reasonable control and further develop the vehicle energy saving potential. The speed prediction and simulation experiment adopts a 1300 s driving cycle without training of any model.

### 4.1. Performance of VSNet

**Figure 9:**
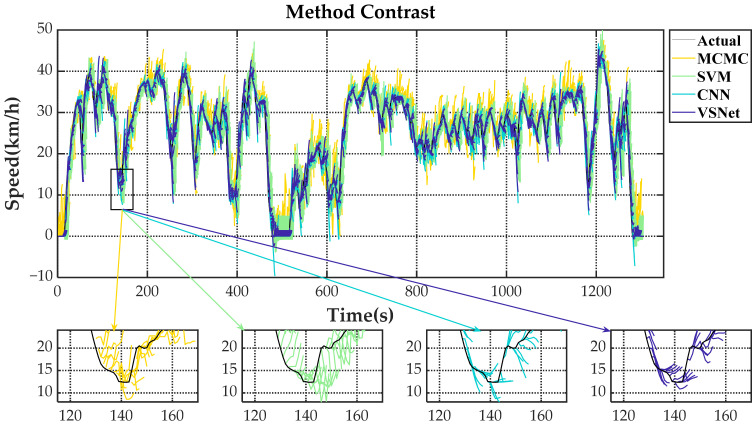
The tail figures of the four methods for the test driving cycles.

**Figure 10:**
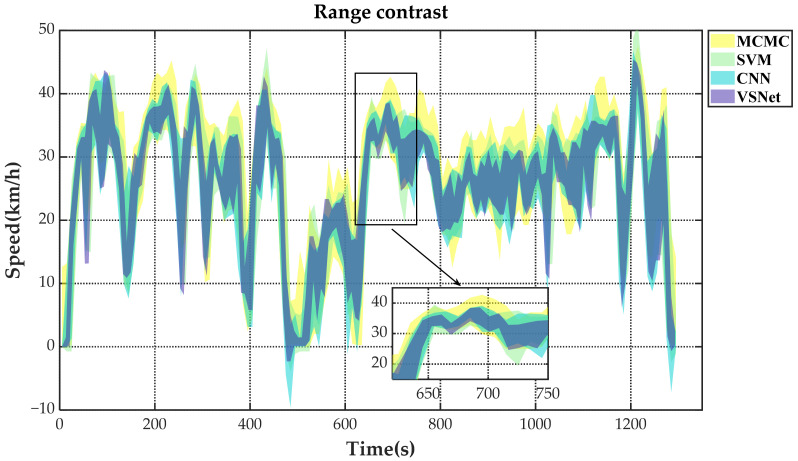
The distribution ranges of the predicted tails of the four methods.

**Figure 11:**
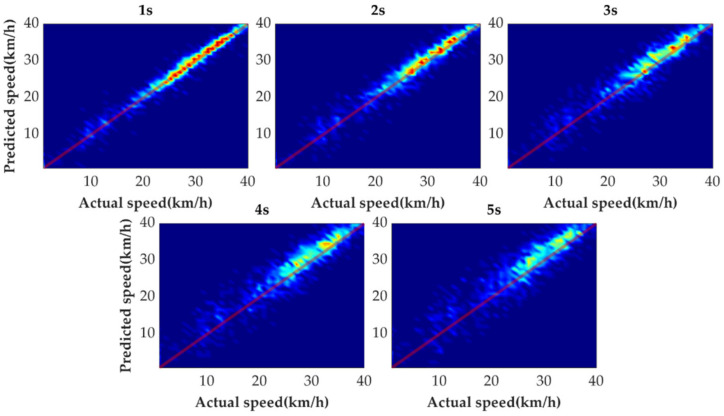
Heat map display of the predicted results of MCMC.

**Figure 12:**
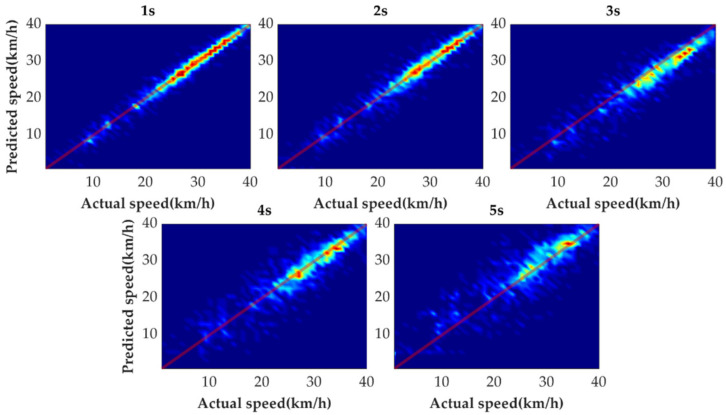
Heat map display of the predicted results of SVM.

**Figure 13:**
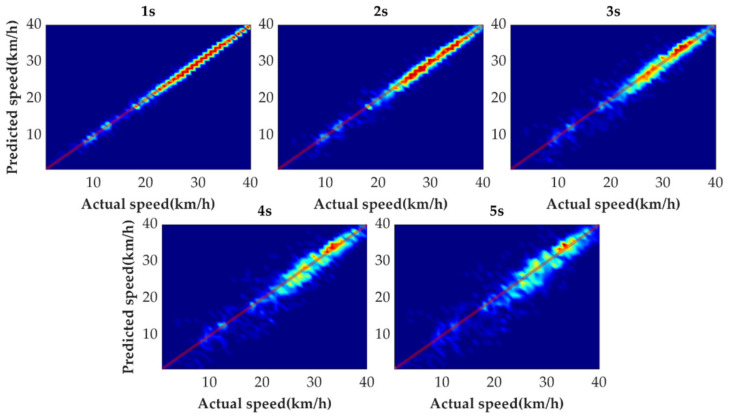
Heat map display of the predicted results of CNN.

**Figure 14:**
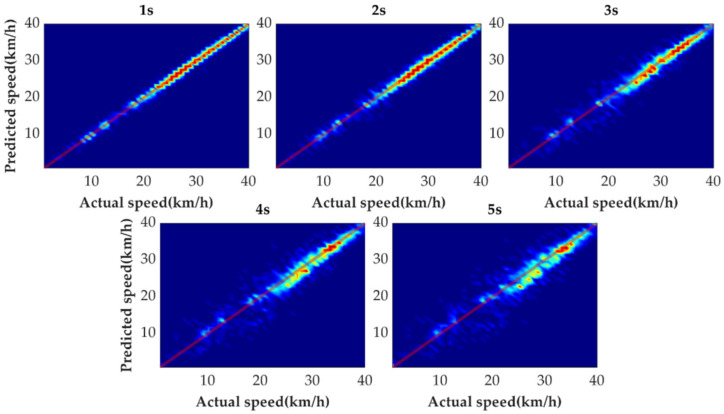
Heat map display of the predicted results of VSNet.

**Figure 15:**
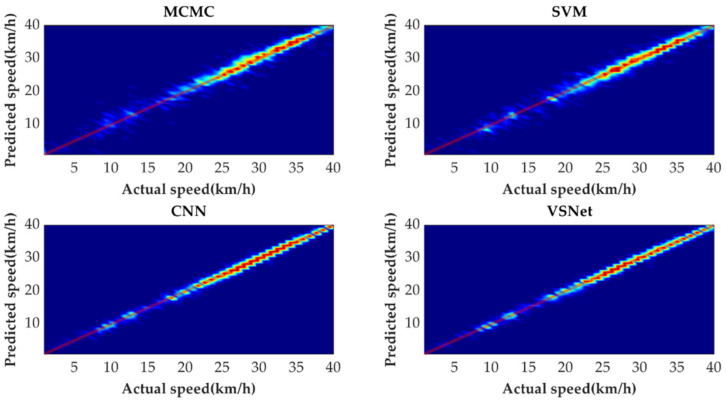
Heat map display of the predicted results for the next 1 s.

**Figure 16:**
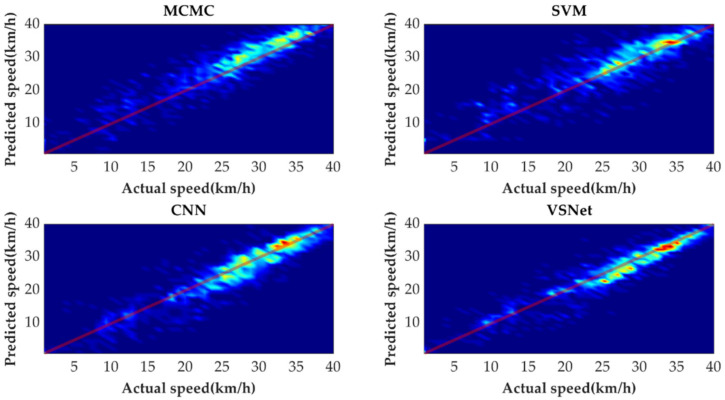
Heat map display of the predicted results for the next 5 s.

**Figure 17:**
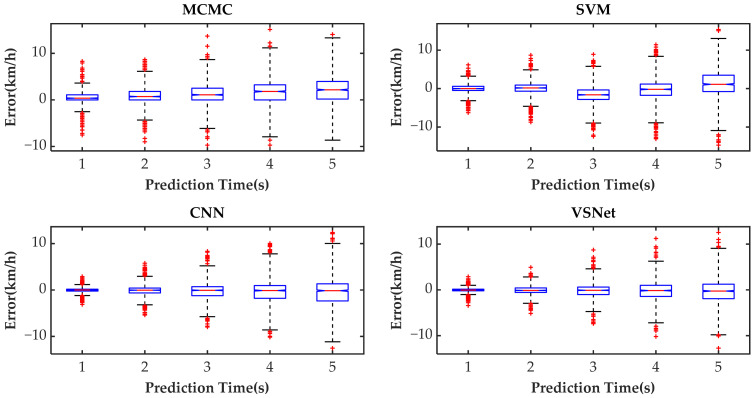
The box plots of the speed prediction errors.

**Figure 18:**
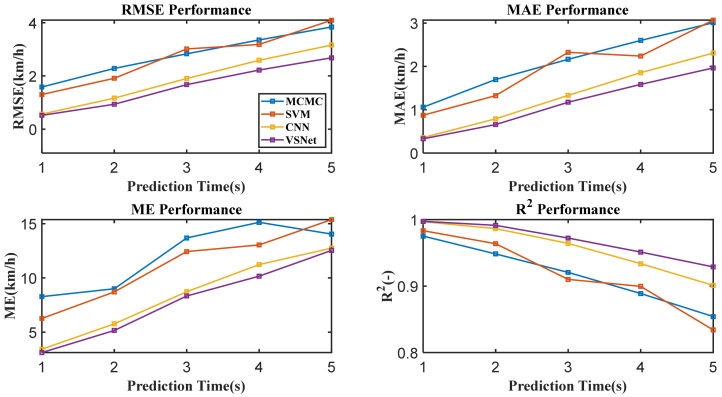
The performance of the four methods.

Figure 9 shows the tail figures of the four methods for the test driving cycle. Figure 10 shows the distribution range of the predicted tails for the different methods. Figure 11, Figure 12, Figure 13 and Figure 14 show the heat maps of the predicted vehicle speed for MCMC, SVM, CNN and VSNet, respectively. Figure 15 and Figure 16 compare the vehicle speed distributions for the first and fifth seconds for the four methods in the form of heat maps. Figure 17 shows the box plots of the speed prediction errors. Figure 18 shows the performance of the four methods.

The black line in Figure 9 is the actual vehicle speed for the test driving cycle. Each colored line is the prediction result of vehicle speed for the next 5 s corresponding to its starting moment. When the colored curves are closer to the black curve, it means the better prediction result. Figure 9 shows that yellow has the widest coverage, followed by green. The blue and dark purple, on the other hand, have a more concentrated distribution of curves, which can only sometimes be distinguished at the turn of the curve. This also means that MCMC has the worst prediction effect, followed by SVM, while CNN and VSNet have better prediction effect. As can be seen from the partial enlargement of Figure 9, MCMC has a strong randomness in the predicted speed change due to the randomness of the process of moving from one state to another in the state space. The process has the non-aftereffect property, which means future evolution does not depend on past evolution under known current conditions. The detail view of SVM has more regular prediction results compared with MCMC because SVM belongs to supervised learning. Supervised learning minimizes the empirical risk and confidence range by finding the structured risk minimum. In this way, the generalization ability of learning machine can be improved and good statistical rules can be obtained. However, the predicted results during acceleration often show a braking state and the predicted results during deceleration are driven. Such opposite prediction affects the performance of energy management strategy. After adopting the deep learning method, the features of input data can be automatically extracted for prediction by combining the vehicle component signal with the vehicle chassis signal and the prediction accuracy is significantly improved. CNN can quickly identify the current vehicle driving state and give the prediction speed of the corresponding state, which leads to a significant reduction of the blue line coverage. However, the prediction results of CNN have a large error when the vehicle state changes. The prediction results of VSNet have higher accuracy compared with those of CNN. The detail view of VSNet shows that the future state of the vehicle can be predicted before the vehicle state changes, which indicates that VSNet can find a more exact mapping relationship between input and output.

Figure 10 shows the distribution of the ranges of predicted tails. The plotting scheme with transparency has been adopted in order to show the overlapping parts more clearly. The intersection of different regions can be identified by color changes. The original colors of the ranges are shown in the legend. From Figure 10, we can get same conclusions as before. MCMC has the widest coverage and SVM is next, which indicates that the predictions are subject to a wide margin of error. The obvious blue area can be found in the process of vehicle state switching, which indicates that the prediction effect of CNN method is still slightly insufficient, while VSNet achieves the best result. A clear blue area can be found in the process of vehicle state switching, indicating that the prediction effect of CNN is still slightly insufficient. VSNet, on the other hand, achieves the best results among all methods.

In order to further evaluate the prediction results reasonably, the errors of each prediction horizon and various prediction methods are compared next. Figure 11, Figure 12, Figure 13, Figure 14, Figure 15 and Figure 16 show the heat maps of the different methods and horizons. The colors in the figure represent density. The closer the color is to red indicates the more consistent prediction results at the actual vehicle speed. The closer the distribution is to the boundary of 45 degrees in the first quadrant, the higher the prediction accuracy is. Firstly, the prediction errors of different time horizons are compared. From Figure 11, Figure 12, Figure 13 and Figure 14, it can be seen that distributions of all prediction methods are gradually deviating from the boundary of 45 degrees as the prediction horizon increases. This represents a gradual increase in prediction error and a significant decrease in prediction accuracy. With the increase of prediction horizon, the driving state of vehicles has more possibilities and the prediction difficulty increases. Moreover, prediction results at the same speed gradually disperse, which leads to a decrease in concentration and even disappearance of the red area. Comparing the high-speed prediction with the low-speed prediction, the results of high-speed prediction of different methods and horizons are better than the results of low-speed prediction. This result arises from the fact that low-speed conditions are mostly caused by environmental constraints, which lead to high complexity and difficulty for prediction. Comparing the different methods, the results of MCMC are slightly higher than the boundary of 45 degrees, especially for predicting the next 3 to 5 s. This indicates that MCMC is more inclined to predict the accelerated results when the state is transferred. The prediction results of SVM at the 3rd second are significantly smaller compared with the actual speed. From the concentration and distribution, it can be seen that the prediction results of CNN and VSNet based on deep learning methods are significantly better than those of SVM and MCMC. This conclusion is easier to draw in Figure 15 and Figure 16. In addition, it can be seen from Figure 17 that VSNet has extremely high accuracy in predicting the speed in the next 1 s and all the data are almost concentrated around the boundary of 45 degrees.

Figure 17 shows the errors distribution of different prediction methods in the form of the box plot. The middle line of the box, which is the median of the data, represents the average level of the predicted errors. The upper and lower limits of the box, respectively, are the upper and lower quartiles of the predicted errors. Therefore, the width of the box reflects to some extent the degree of fluctuation of the data. The whiskers extend to the most extreme data points not considered outliers, and the outliers are plotted individually using the ‘+’ symbol. All bounds except the median are gradually moved away from 0 km/h as the predicted horizon increases. As the range between the lower and upper boundaries is wider, the outliers beyond the range also gradually decrease. Since there are more acceleration states than braking states during the historical state transfer of the vehicle, the median MCMC gradually increases as the prediction horizon increases. The median of SVM fluctuates around 0 km/h with the third second being the most obvious. The medians of CNN and VSNet are approximately maintained at 0 km/h. The 25th and 75th percentiles of VSNet are obviously closer to 0 km/h, which proves that VSNet has better prediction effect.

Figure 18 shows the RMSE, Mean Absolute Error (MAE), Maximum Absolute Error (ME) and R Squared (R^2^) for the different methods at different horizons. It can be observed intuitively from the figure that VSNet outperforms the other three methods in terms of MAE, RMSE, ME and R2 in all horizons.

### 4.2. Simulation

In this paper, the vehicle model under the Matlab/Simulink platform is established based on the vehicle configuration in Section 3.1. The vehicle model is equipped with different energy management strategies and simulated for the driving cycle in Section 4.1. The different energy management strategies are the rule-based energy management strategy, the global-optimization-based energy management strategy, and the MPC-based energy management strategies adopting the vehicle speed prediction methods of MCMC, SVM, CNN, VSNet and the known cycle. The known cycle method achieves 100% prediction accuracy by taking the known information of the test driving cycle as the result of speed prediction. The simulation results are analyzed in equivalent fuel consumption, fuel consumption, SOC and operating points of components. The effectiveness of the MPC-based energy management strategy is verified, and the relationship between the speed prediction accuracy and the optimization effect of strategies is explored. The initial SOC set for the simulation is 28% and the vehicle is driven in Charge Depleting (CD) mode during the first half of the simulation. As the power is consumed the vehicle enters Charge Sustaining (CS) mode. The entire process is based on the vehicle speed and the overall vehicle demand power to choose single power source traction mode, hybrid traction mode or charging mode, maintaining the SOC near 25%. The single traction mode, hybrid traction mode or charging mode is selected according to the overall vehicle demand power and SOC to maintain SOC around 25%.

**Figure 19:**
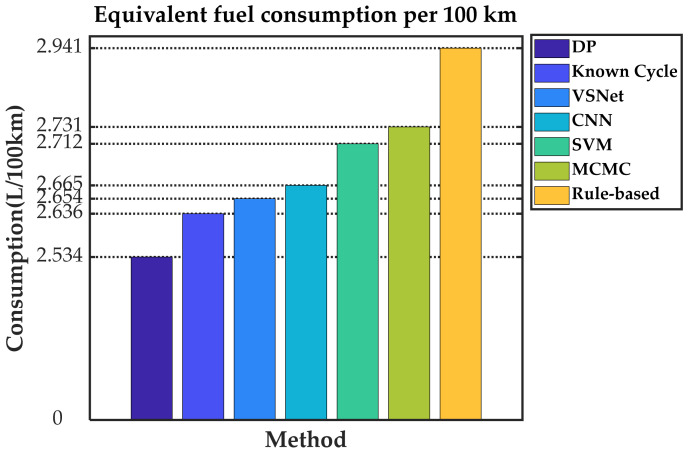
The equivalent fuel consumption for the simulations with different energy management strategies.

**Figure 20:**
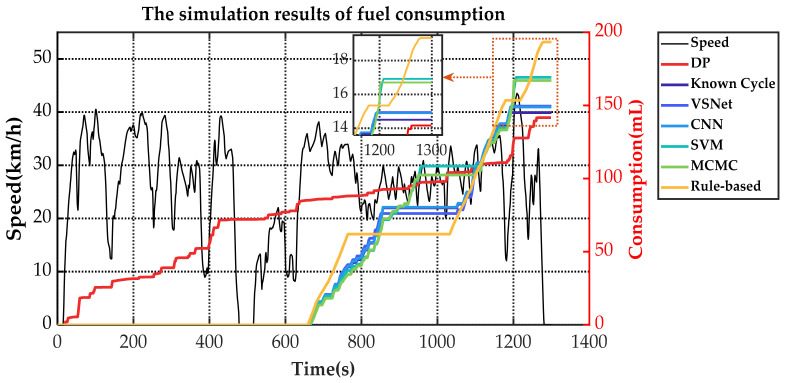
The fuel consumption during the simulations with different energy management strategies.

**Figure 21:**
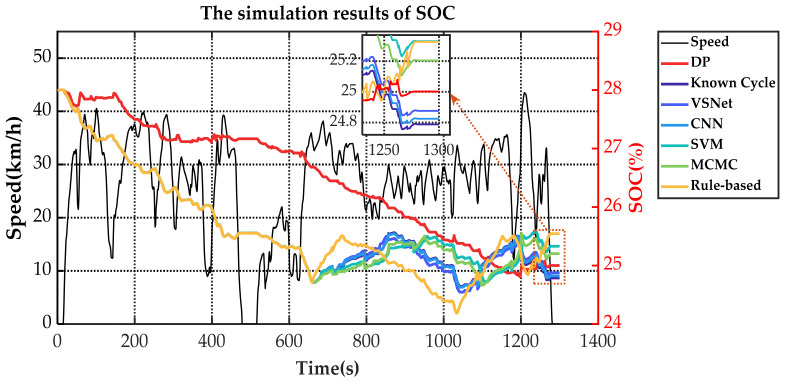
The SOC during the simulations with different energy management strategies.

**Figure 22:**
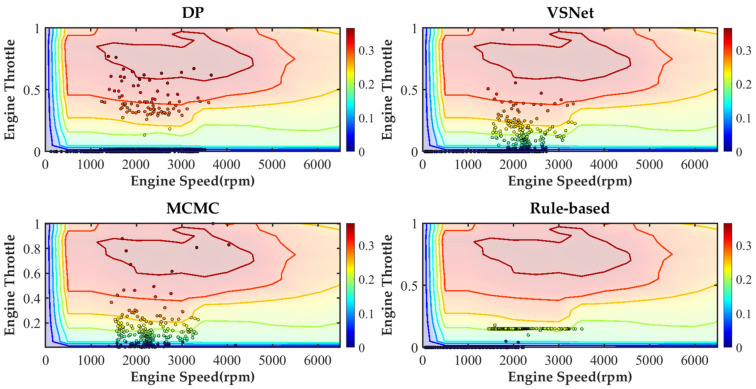
The distribution of engine operating points under various strategies.

**Figure 23:**
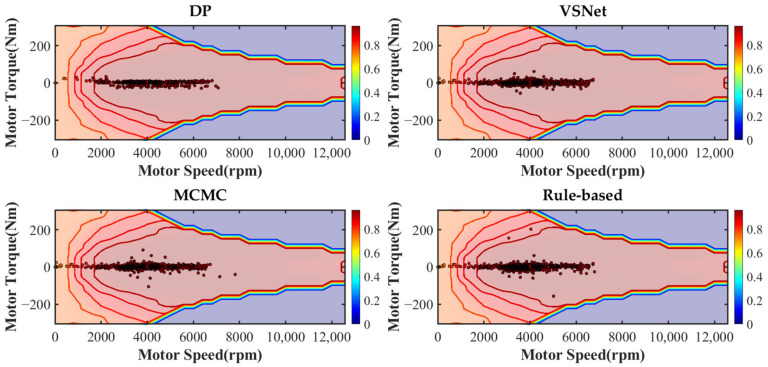
The distribution of motor operating points under various strategies.

Figure 19 and Table 3 show the equivalent fuel consumption for the simulations with different energy management strategies. Figure 20 shows the fuel consumption during the simulations with different energy management strategies. Figure 21 shows the SOC during the simulations with different energy management strategies. Figure 22 shows the distribution of engine operating points. Figure 23 shows the distribution of motor operating points.

As can be seen in Figure 19, optimization-based energy management strategies have lower equivalent fuel consumption compared with the rule-based energy management strategy, regardless of the optimization method adopted. As a criterion of optimization effectiveness, DP has the best economy with an equivalent fuel consumption of only 2.534 L/100 km. In the terms of instantaneous-optimization-based energy management strategies, known-cycle-based MPC can be optimized according to real future driving conditions and achieves an equivalent fuel consumption of 2.636 L/100 km with 4.03% more than DP. MCMC-based MPC achieves the highest equivalent fuel consumption in the MPC methods with 7.77% more than DP and 7.14% less than rule-based because of its fluctuating predicted speed. VSNet, which has the best speed prediction capability, achieved the best MPC-based optimization results except for known cycle. The equivalent fuel consumption of VSNet-based MPC is 2.654 L/100 km, which is 4.74% less than DP and only 0.018 L/100 km higher than known-cycle-based MPC.

The changes of fuel consumption in Figure 20 show that the DP starts the engine frequently to keep the fuel consumption slowly rising. CNN-based MPC, VSNet-based MPC and known-cycle-based MPC approximately maintain the same operating period and fuel consumption. The rule-based energy management strategy obtains the highest energy consumption of all methods. Since not optimized for operating points of the engine, it has an approximately constant fuel consumption rate. Combined with Figure 21, results show that DP often starts the engine to assist the motor in sharing the vehicle demand power. Therefore, its SOC changes are also relatively flat and nearly linearly decreasing. For MPC, the SOC fluctuation range is roughly the same for all five methods. The difference is that the fuel consumption and SOC of MCMC-based MPC and SVM-based MPC increase more gently and charge more slowly. CNN-based MPC and VSNet-based MPC are closer to known-cycle-based MPC. The rule-based energy management strategy has the maximum charging power. In the terminated state, CNN-based MPC, VSNet-based MPC and known-cycle-based MPC maintain similar SOC. Rule-based energy management strategy, MCMC-based MPC and SVM-based MPC have higher SOC and less electricity consumption at the cost of greater fuel consumption.

In order to further analyze the working condition of components, distributions of engine operating points and motor operating points under DP, VSNet-based MPC, MCMC-based MPC and rule-based energy management strategy are compared. From Figure 22, points represent the engine operating points and colors represent the efficiency of the engine. In the results of DP, the engine adopts a high power to drive the motor to charge the battery each time it is turned on. The large throttle percentage increases the engine loading rate and improves the engine efficiency. MPC does not select engine operating points of high power in order to obtain the smallest objective function in the predicted horizon. The operating points of the motor are only partially distributed in the high efficiency area. VSNet-based MPC relies on high accuracy prediction of vehicle speed to obtain a better distribution of engine operating points compared with MCMC-based MPC. The rule-based energy management strategy has poor regulation capability and more concentrated distribution.

From Figure 23, points represent the motor operating points and colors represent the efficiency of the motor. Observe the distribution, as most of operating points are concentrated in the high-efficiency region, with DP being the most concentrated and VSNet being second. The least effective is the rule-based energy management strategy. Due to an improper power distribution ratio, the motor sometimes undertakes too much power. Some motor operating points have been distributed to the area of low efficient.

## 5. Conclusion and Future Outlook

In this paper, an architecture for the deep learning-based neural network called VSNet is constructed. VSNet is able to predict future vehicle speed just by self-historical data. A fake image consisting of 15 vehicle signals for the past 15 s is input into VSNet to predict the vehicle speed for the next 5 s. Representative methods from stochastic prediction, machine learning and deep learning are selected for comparison with VSNet. We employ all methods for prediction for a driving cycle that is not involved in any model training. From the prediction results, it can be concluded that RMSE, MAE, ME and R^2^ of VSNet are better than the other methods. An MPC energy management strategy based on the speed prediction method is also constructed for simulation and analysis. The simulation results can be summarized that the power optimization effect of MPC is positively correlated with the speed prediction accuracy. With the increase of prediction accuracy of vehicle speed, the difference of MPC compared with DP can be reduced from 7.77% to 4.03%, and the simulation results are closer to the results of DP in terms of fuel consumption, electrical consumption and power distribution.

In this paper, we design a vehicle speed prediction method with high accuracy for power distribution of multi-power system. In the future, we will build an energy management strategy for complex driving condition based on fused multi-sensor information.

## Figures and Tables

**Table 1:** Pearson correlation coefficient of selected signals.

CAN Bus Signal	Pearson Correlation Coefficient
Vehicle Speed (km/h)	1
Powertrain Speed (rpm)	0.971832
Engine Speed (rpm)	0.832933
Generator Speed (rpm)	0.83258
Generator Efficiency	0.786406
Motor Speed (rpm)	0.768914
Motor Efficiency	0.707602
Engine Fuel Consumed (gram)	0.686369
Engine Fuel rate (g/kwh)	0.657577
Engine On	0.581088
Clutch On	0.45993
Engine Torque (Nm)	0.397152
Engine Throttle	0.36374
Regen Brake	0.250294
Battery Current (A)	0.225507

**Table 2:** Vehicle and component parameters in the studied vehicle.

Component	Parameter	Value	Unit
Vehicle	Vehicle weight	1200	kg
	Tire radius	0.323	m
	Final drive ratio	4.021	/
Engine	Maximum torque	165	Nm
	Maximum power	105@6500	kW@rpm
Motor	Maximum torque	307	Nm
	Maximum power	126@12,584	kW@rpm
Battery	Rated capacity	20.8	Ah
	Rated voltage	366	V
Gear	First	3.527	/
	Second	2.025	/
	Third	1.382	/
	Fourth	1.058	/
	Fifth	0.958	/

**Table 3:** Simulation results.

Method	Fuel Consumption (mL)	Final SOC (%)	Equivalent Fuel Consumption (L/100 km)	Increased Equivalent Fuel Consumption Compared with DP (%)
DP	141.64	25.00	2.534	/
Known Cycle	145.05	24.79	2.636	4.03
VSNet	149.55	24.88	2.654	4.74
CNN	148.93	24.83	2.665	5.17
SVM	169.17	25.33	2.712	7.02
MCMC	167.03	25.20	2.731	7.77
Rule-based	193.35	25.32	2.941	16.06

## Data Availability

Not applicable.

## References

[1] Gan L., Lv W., Chen Y. (2021). Capital structure adjustment speed over the business cycle. Financ. Res. Lett..

[2] Sokolov-Mladenovic S., Milovancevic M., Mladenovic I., Alizamir M. (2016). Economic growth forecasting by artificial neural network with extreme learning machine based on trade, import and export parameters. Comput. Hum. Behav..

[3] De Moura J., Yang J., Butt S.D. (2021). Physics-Based Rate of the Penetration Prediction Model for Fixed Cutter Drill Bits. J. Energy Resour. Technol..

[4] Han S., Zhang F., Xi J., Ren Y., Xu S. Short-term Vehicle Speed Prediction Based on Convolutional Bidirectional LSTM Networks. Proceedings of the 2019 IEEE Intelligent Transportation Systems Conference (ITSC).

[5] Jinquan G., Hongwen H., Jianwei L., Qingwu L. (2021). Real-time Energy Management of Fuel Cell Hybrid Electric Buses: Fuel Cell Engines Friendly Intersection Speed Planning. Energy.

[6] Ma D., Song X., Li P. (2021). Daily Traffic Flow Forecasting Through a Contextual Convolutional Recurrent Neural Network Modeling Inter- and Intra-Day Traffic Patterns. IEEE Trans. Intell. Transp. Syst..

[7] Li Y.F., Chen M.N., Zhao W.Z. (2019). Investigating long-term vehicle speed prediction based on BP-LSTM algorithms. IET Intell. Transp. Syst..

[8] Ibrahim A., Goswami D., Li H., Soroa I.M., Basten T. (2021). Multi-layer multi-rate model predictive control for vehicle platooning under IEEE 802.11p. Transp. Res. Part C Emerg. Technol..

[9] Wang X., Chen J., Quan S., Wang Y.-X., He H. (2020). Hierarchical model predictive control via deep learning vehicle speed predictions for oxygen stoichiometry regulation of fuel cells. Appl. Energy.

[10] Pei J., Su Y., Zhang D., Qi Y., Leng Z. (2020). Velocity forecasts using a combined deep learning model in hybrid electric vehicles with V2V and V2I communication. Sci. China Technol. Sci..

[11] Yang C., Zha M., Wang W., Liu K., Xiang C. (2020). Efficient energy management strategy for hybrid electric vehicles/plug-in hybrid electric vehicles: Review and recent advances under intelligent transportation system. IET Intell. Transp. Syst..

[12] Li M., He H., Feng L., Chen Y., Yan M. (2020). Hierarchical predictive energy management of hybrid electric buses based on driver information—ScienceDirect. J. Clean. Prod..

[13] Huang Y., Wang H., Khajepour A., He H., Ji J. (2017). Model predictive control power management strategies for HEVs: A review. J. Power Sources.

[14] Mirbaha B., Saffarzadeh M., Beheshty S.A., Aniran M., Yazdani M., Shirini B. (2017). Predicting Average Vehicle Speed in Two Lane Highways Considering Weather Condition and Traffic Characteristics.

[15] Maji A., Sil G., Tyagi A. (2018). 85th and 98th Percentile Speed Prediction Models of Car, Light, and Heavy Commercial Vehicles for Four-Lane Divided Rural Highways. J. Transp. Eng. Part A Syst..

[16] Li L., Coskun S., Zhang F., Langari R., Xi J. (2019). Energy Management of Hybrid Electric Vehicle Using Vehicle Lateral Dynamic in Velocity Prediction. IEEE Trans. Veh. Technol..

[17] Ma Z.Y., Zhang Y.Q., Yang J. (2016). Velocity and normal tyre force estimation for heavy trucks based on vehicle dynamic simulation considering the road slope angle. Veh. Syst. Dyn..

[18] Shin J., Sunwoo M. (2018). Vehicle Speed Prediction Using a Markov Chain with Speed Constraints. IEEE Trans. Intell. Transp. Syst..

[19] Karbowski D., Kim N., Rousseau A. Route-Based Online Energy Management of a PHEV and Sensitivity to Trip Prediction. Proceedings of the 2014 IEEE Vehicle Power and Propulsion Conference (VPPC).

[20] Li Y., Chen M., Lu X., Zhao W. (2018). Research on optimized GA-SVM vehicle speed prediction model based on driver-vehicle-road-traffic system. Sci. China.

[21] Hosseini M.K., Talebpour A. (2019). Traffic Prediction using Time-Space Diagram: A Convolutional Neural Network Approach. J. Transp. Res. Board.

[22] Ma X., Tao Z., Wang Y., Yu H., Wang Y. (2015). Long short-term memory neural network for traffic speed prediction using remote microwave sensor data. Transp. Res. Part C Emerg. Technol..

[23] Yeon K., Min K., Shin J., Sunwoo M., Han M. (2019). Ego-Vehicle Speed Prediction Using a Long Short-Term Memory Based Recurrent Neural Network. Int. J. Automot. Technol..

[24] Chi J.T., Ipsen I.C. (2021). Multiplicative perturbation bounds for multivariate multiple linear regression in Schatten p-norms. Linear Algebra Appl..

[25] Hill G., Blythe P.T., Higgins C. Deviations in Markov chain modeled electric vehicle charging patterns from real world data. Proceedings of the 15th International IEEE Conference on Intelligent Transportation Systems 2012.

[26] Hemi H., Ghouili J., Cheriti A. (2015). Combination of Markov chain and optimal control solved by Pontryagin’s Minimum Principle for a fuel cell/supercapacitor vehicle. Energy Convers. Manag..

[27] Sah S. (2020). Machine Learning: A Review of Learning Types. Preprints.

[28] Cuenca L.G., Sanchez-Soriano J., Puertas E., Andrés J.F., Aliane N. (2019). Machine Learning Techniques for Undertaking Roundabouts in Autonomous Driving. Sensors.

[29] Nguyen H., Kieu L.M., Wen T., Cai C. (2018). Deep learning methods in transportation domain: A review. IET Intell. Transp. Syst..

[30] Shrestha A., Mahmood A. (2019). Review of Deep Learning Algorithms and Architectures. IEEE Access.

[31] Wang Z.J., Turko R., Shaikh O., Park H., Das N., Hohman F., Kahng M., Chau D.H.P. (2020). CNN Explainer: Learning Convolutional Neural Networks with Interactive Visualization. IEEE Trans. Vis. Comput. Graph..

[32] Wang C., Hou Y., Barth M. (2019). Data-Driven Multi-Step Demand Prediction for Ride-Hailing Services Using Convolutional Neural Network.

[33] Loaiza F.A., Herrera J., Mantilla S.C.L. Using a Separable Convolutional Neural Network for Large-Scale Transportation Network Speed Prediction. Proceedings of the 10th International Conference on Predictive Models in Software Engineering 2018.

[34] Ma X., Dai Z., He Z., Ma J., Wang Y., Wang Y. (2017). Learning Traffic as Images: A Deep Convolutional Neural Network for Large-Scale Transportation Network Speed Prediction. Sensors.

[35] Qi X., Zheng X., Chen Q. (2020). A short term load forecasting of integrated energy system based on CNN-LSTM. E3S Web of Conferences.

[36] Hochreiter S., Schmidhuber J. (1997). Long Short-Term Memory. Neural Computation.

[37] Pérez-Ortiz J.A., Gers F.A., Eck D., Schmidhuber J. (2003). Kalman filters improve LSTM network performance in problems unsolvable by traditional recurrent nets. Neural Netw..

[38] Graves A., Fernández S., Gomez F., Schmidhuber J. Connectionist temporal classification: Labelling unsegmented sequence data with recurrent neural networks. Proceedings of the 23rd International Conference on Machine Learning.

[39] Graves A., Jaitly N., Mohamed A.-R. Hybrid speech recognition with Deep Bidirectional LSTM. Proceedings of the 2013 IEEE Workshop on Automatic Speech Recognition and Understanding.

[40] Feng W., Zhu Q., Zhuang J., Yu S. (2018). An expert recommendation algorithm based on Pearson correlation coefficient and FP-growth. Clust. Comput..

[41] Borhan H., Vahidi A., Phillips A.M., Kuang M.L., Kolmanovsky I.V., Di Cairano S. (2011). MPC-Based Energy Management of a Power-Split Hybrid Electric Vehicle. IEEE Trans. Control Syst. Technol..

[42] Karim S.A.A., Ismail M.T., Hasan M.K., Sulaiman J. (2018). Data interpolation using Runge Kutta method. Proceedings of the 25th National Symposium on Mathematical Sciences (Sksm25): Mathematical Sciences as the Core of Intellectual Excellence.

[43] Andersson J.A.E., Gillis J., Horn G., Rawlings J.B., Diehl M. (2019). CasADi: A software framework for nonlinear optimization and optimal control. Math. Program. Comput..

[44] Yamashita H., Yabe H. (2010). Local and superlinear convergence of a primal-dual interior point method for nonlinear semidefinite programming. Math. Program..

